# Supplementary material for: Assessing clinical quality performance and staffing capacity differences between urban and rural Health Resources and Services Administration-funded health centers in the United States: A cross sectional study
Source: PLoS One. 2020 Dec 8;15(12):e0242844. doi: 10.1371/journal.pone.0242844 (PMC7723285; doi:10.1371/journal.pone.0242844)
Supplement: S1 Checklist — (DOC) [file pone.0242844.s001.doc]

STROBE Statement—Checklist of items that should be included in reports of ***cross-sectional studies***

|  | Item No | Recommendation | Location |
| --- | --- | --- | --- |
| **Title and abstract** | 1 | (*a*) Indicate the study’s design with a commonly used term in the title or the abstract | **Title**  Assessing clinical quality performance and staffing capacity differences between urban and rural Health Resources and Services Administration-funded health centers in the United States: A cross sectional study  **Abstract (methods and findings)**  **Methods and Findings:** We used the 2017 Uniform Data System to examine performance on clinical quality measures between urban and rural HCs (n=1,373). We used generalized linear regression models to control for confounding factors. After adjusting for potential confounders, we found on par performance between urban and rural HCs in all but one clinical quality measure. Rural HCs had lower rates of linking patients newly diagnosed with HIV to care (74% [95% CI: 69%, 80%] vs. 83% [95% CI: 80%, 86%]). We identified control variables that systematically accounted for eliminating urban vs. rural differences in performance on clinical quality measures. We also found that both urban and rural HCs had some clinical quality performance measures that were lower than available national benchmarks. Main limitations included potential discrepancy of urban or rural designation across all HC sites within a HC organization. |
|  |  | (*b*) Provide in the abstract an informative and balanced summary of what was done and what was found | **Abstract (methods and findings)**  **Methods and Findings:** We used the 2017 Uniform Data System to examine performance on clinical quality measures between urban and rural HCs (n=1,373). We used generalized linear regression models to control for confounding factors. After adjusting for potential confounders, we found on par performance between urban and rural HCs in all but one clinical quality measure. Rural HCs had lower rates of linking patients newly diagnosed with HIV to care (74% [95% CI: 69%, 80%] vs. 83% [95% CI: 80%, 86%]). We identified control variables that systematically accounted for eliminating urban vs. rural differences in performance on clinical quality measures. We also found that both urban and rural HCs had some clinical quality performance measures that were lower than available national benchmarks. Main limitations included potential discrepancy of urban or rural designation across all HC sites within a HC organization. |
| Introduction | | |  |
| Background/rationale | 2 | Explain the scientific background and rationale for the investigation being reported | **Introduction, paragraphs 1-3**  An estimated 72 percent of the land area in the United States is considered rural and 14 percent of the population or 46.1 million people live in rural areas.[1] Evidence indicates significant and persistent rural disparities in quality of care. Rural areas (micropolitan and noncore) were found to have worse quality of care based on performance on 250 quality indicators examined compared to large suburban areas.[2] In addition, these disparities have remained the same for over one third or worsened for about one in ten of these indicators from 2000 to 2015.[2] The indicators with worse quality of care constituted about a third of the 26 effective treatment indicators, a third to half of the care coordination indicators, and a quarter to one third of the 18 access to care indicators in rural areas.[2] These disparities co-occur with variations in sociodemographics and disparities in health status.[3-6] Nationally, rural populations are more socioeconomically vulnerable, including more often without high school education (16% vs. 13%), more often low-income (38% vs. 33% under federal poverty guidelines), Medicaid insured (24% vs. 22%), less often employed (56% vs. 58%), less often female (48% vs 50%), and more often in poorer health (13% vs. 9%) compared to urban areas.[7-9] In addition, 85% of rural counties in the United States were persistently considered primary care Health Professional Shortage Areas at least once from 1996 to 2005, which impacts access to care and might impact clinical quality measure performance.[10, 11]  Health centers funded by the Health Resources and Services Administration (HRSA) (referred to as HCs hereon) have a significant presence in rural areas and may be the only providers in some rural locations.[3] In 2017, about 44% of the 1,373 HCs served rural populations, operated more than 4,400 sites, and collectively provided care through 35 million visits to more than 27 million total patients.[12] Earlier studies of HCs indicated that rural HC patients were more often older, female, white, poor, uninsured, obese, in poor health, and with activity limitations compared to the general rural population.[3] In addition, rural HCs had lower staffing supply for primary, mental health, and dental care providers than urban settings.[3, 4, 6, 13, 14] Strategic quality improvement initiatives over the years have improved provider recruitment and retention in rural areas and may have reduced disparities in access to care and quality of care.[15, 16] In fact, past research assessing performance on clinical quality at HCs has shown that rural HCs performed well in prenatal care outcomes, cervical cancer screening, and childhood immunization rates.[3, 17, 18]  Existing data on urban/rural disparities in quality of care among HCs is limited, and a gradual decline in the size of rural populations may have influenced previous understanding of this issue.[1] A comprehensive assessment of the clinical quality of HCs in rural communities is necessary to identify areas in need of improvement and reduce missed opportunities in addressing rural disparities in quality of care. |
| Objectives | 3 | State specific objectives, including any prespecified hypotheses | **Introduction, paragraph 3**  To address this gap, we examined differences in performance on clinical quality measures between rural and urban HCs, controlling for differences in characteristics of these organizations as well as other contextual factors that might impact performance on these measures. We hypothesized that rural HCs performed as well as urban HCs because of the emphasis HRSA has placed on improving access to quality health care services. HRSA has supported quality improvement by requiring reporting on performance, incentivizing improvement and meeting national benchmarks of performance and providing financial and technical support to improve performance.[19-21] Our study aimed to highlight the contributions of rural HCs in reducing disparities in quality of care and identify programmatic improvements that could maximize these contributions. |
| Methods | | |  |
| Study design | 4 | Present key elements of study design early in the paper | **Introduction, paragraph 3**  To address this gap, we examined differences in performance on clinical quality measures between rural and urban HCs, controlling for differences in characteristics of these organizations as well as other contextual factors that might impact performance on these measures.  **Methods, paragraph 1-2 “Data and sample” subsection**  **Data and sample**. For this cross-sectional study, we used data from the 2017 Uniform Data System (UDS) reported by all HRSA-funded HCs on organizational characteristics and clinical quality measures. UDS is an administrative data source maintained by HRSA to monitor the Health Center Program and provide information to stakeholders. HRSA-funded HCs and other entities receiving federal funding authorized under Section 330 of the Public Health Service Act are required to report UDS data. UDS captures aggregate information at the organization level rather than individual delivery sites that operate within the organization.[19]  We merged the UDS data with the latest available relevant data from the 2016 Area Health Resource File (AHRF). AHRF is a publicly available dataset maintained by HRSA. It compiles information from over 50 sources to provide county-level data on population characteristics, health workforce availability, health care utilization, and health facilities.[22] We merged these data using the Federal Information Process Standards (FIPS) code associated with the address of the HC organization. If HCs were present in multiple counties, we merged the data for the county where the largest share of HC patients lived. We included all HCs that reported serving patients in 2017 for a total analytic sample of 1,373. |
| Setting | 5 | Describe the setting, locations, and relevant dates, including periods of recruitment, exposure, follow-up, and data collection | **Methods, paragraph 1-2 “Data and sample” subsection**  **Data and sample.** For this cross-sectional study, we used data from the 2017 Uniform Data System (UDS) reported by all HRSA-funded HCs on organizational characteristics and clinical quality measures. UDS is an administrative data source maintained by HRSA to monitor the Health Center Program and provide information to stakeholders. HRSA-funded HCs and other entities receiving federal funding authorized under Section 330 of the Public Health Service Act are required to report UDS data. UDS captures aggregate information at the organization level rather than individual delivery sites that operate within the organization.[19]  We merged the UDS data with the latest available relevant data from the 2016 Area Health Resource File (AHRF). AHRF is a publicly available dataset maintained by HRSA. It compiles information from over 50 sources to provide county-level data on population characteristics, health workforce availability, health care utilization, and health facilities.[22] We merged these data using the Federal Information Process Standards (FIPS) code associated with the address of the HC organization. If HCs were present in multiple counties, we merged the data for the county where the largest share of HC patients lived. We included all HCs that reported serving patients in 2017 for a total analytic sample of 1,373. |
| Participants | 6 | (*a*) Give the eligibility criteria, and the sources and methods of selection of participants | **Methods, paragraph 1-2 “Data and sample” subsection**  **Data and sample**. For this cross-sectional study, we used data from the 2017 Uniform Data System (UDS) reported by all HRSA-funded HCs on organizational characteristics and clinical quality measures. UDS is an administrative data source maintained by HRSA to monitor the Health Center Program and provide information to stakeholders. HRSA-funded HCs and other entities receiving federal funding authorized under Section 330 of the Public Health Service Act are required to report UDS data. UDS captures aggregate information at the organization level rather than individual delivery sites that operate within the organization.[19]  We merged the UDS data with the latest available relevant data from the 2016 Area Health Resource File (AHRF). AHRF is a publicly available dataset maintained by HRSA. It compiles information from over 50 sources to provide county-level data on population characteristics, health workforce availability, health care utilization, and health facilities.[22] We merged these data using the Federal Information Process Standards (FIPS) code associated with the address of the HC organization. If HCs were present in multiple counties, we merged the data for the county where the largest share of HC patients lived. We included all HCs that reported serving patients in 2017 for a total analytic sample of 1,373. |
| Variables | 7 | Clearly define all outcomes, exposures, predictors, potential confounders, and effect modifiers. Give diagnostic criteria, if applicable | **Methods, paragraph 3-5 “Dependent Variables” and “Independent Variables” subsection**  Dependent Variables. We studied clinical quality measures that HCs are required to report. The majority of these measures were standard quality metrics and concordant with Centers for Medicare and Medicaid Services guidelines and electronically specified for automated reporting by HCs (electronic clinical quality measures).[23] These measures had national benchmarks included in the 2017 Healthcare Effectiveness Data and Information Set (HEDIS) for Medicaid Managed Care patients.[24, 25] A total of 15 measures were included in our analyses, with seven measures examining prevention-related performance, five measures associated with care management, and three measures assessing clinical outcomes. All measures were treated as continuous variables. The seven prevention measures included (1) percent of children 2 years of age immunized in accordance with recommended guidelines from the Advisory Committee on Immunization Practices, (2) patients with Pap tests as recommended (percentage of women ages 21-64 years who had cervical cytology performed every three years, and women ages 30-64 who had cervical cytology/human papillomavirus co-testing performed every five years), (3) patients ages 50–75 years old who received appropriate colorectal cancer screening, (4) adult patients 18 years and older screened for tobacco use and cessation counseling and intervention, (5) patients 12 years or older who were routinely screened for depression and received a follow-up plan, (6) adolescents and children ages 3-17 years old with documented body mass index (BMI) percentile and counseling on nutrition and exercise, and (7) percent of adults 18 years and older with documented BMI and follow-up plan. The five measures examining aspects of care management included: (1) patients ages 5-64 years old with persistent asthma who received pharmacological therapy, (2) patients with coronary artery disease and high lipid levels that received lipid lowering therapy, (3) patients with ischemic vascular disease who used aspirin or another antithrombotic drug, (4) patients seen for follow-up care within 90 days of initial HIV diagnosis, and (5) pregnant women who received prenatal care in their first trimester. Three measures examined outcomes of care, including (1) percent of patients with diabetes whose hemoglobin A1c level was greater than 9% at the last measurement for the year (poorly controlled), (2) patients with diagnosed hypertension whose blood pressure was below 140/90 (controlled) at last reading during the year, and (3) patients born to health center patients whose birthweight was below normal (2,500 grams).  Independent Variables. The primary variable of interest was urban/rural status. HCs self-reported this status for the organization and used the same designation for all delivery sites. In some instances, HCs had several service delivery sites across both urban and rural areas, which resulted in some misclassification. We controlled for several HC organizational characteristics, patient characteristics, and contextual variables to account for any potential confounding among HC and local area factors. The HC organizational characteristics controlled for included the organizational size indicated by the number of sites and number of patients seen in 2017. We further controlled for patient demographic and health characteristics including percent of patients ages 0–17 and ages 65 or older, patients who were racial/ethnic minorities, patients who communicated with the provider in a language other than English, patients with heart related disease, patients with diabetes or endocrine disease, patients with respiratory disease, patients with HIV, and prenatal care patients who delivered during the year. These variables controlled for HC case mix and challenges to care outcomes.  We next controlled for primary care and other care capacity and service availability using several indicators. These included the ratio of HC patients per each full-time equivalent (FTE) primary care provider (PCPs include physicians, nurse practitioners, and physician assistants) and ratio of FTE nurses per PCP, the ratio of mental health providers (psychiatrists, psychologists, licensed clinical social workers, other licensed mental health providers) per 5,000 patients, dental providers (dentists and hygienists) per 2,500 patients, enabling service staff (case managers, transportation, and translation staff) per 5,000 patients, and an index of number of services available in addition to medical care. We also included financial resource indicators, including per capita total revenues to measure success in generating revenues and percent of grant revenues from the Section 330 grants to measure success in fundraising. The contextual control variables, extracted from AHRF, included the ratio of PCP per 5,000 individuals in the county, the percentage of individuals below the federal poverty guideline, and the percentage of minorities in the county. |
| Data sources/ measurement | 8* | For each variable of interest, give sources of data and details of methods of assessment (measurement). Describe comparability of assessment methods if there is more than one group | **Methods, paragraph 1-2 “Data and sample” subsection**  **Data and sample.** For this cross-sectional study, we used data from the 2017 Uniform Data System (UDS) reported by all HRSA-funded HCs on organizational characteristics and clinical quality measures. UDS is an administrative data source maintained by HRSA to monitor the Health Center Program and provide information to stakeholders. HRSA-funded HCs and other entities receiving federal funding authorized under Section 330 of the Public Health Service Act are required to report UDS data. UDS captures aggregate information at the organization level rather than individual delivery sites that operate within the organization.[19]  We merged the UDS data with the latest available relevant data from the 2016 Area Health Resource File (AHRF). AHRF is a publicly available dataset maintained by HRSA. It compiles information from over 50 sources to provide county-level data on population characteristics, health workforce availability, health care utilization, and health facilities.[22] We merged these data using the Federal Information Process Standards (FIPS) code associated with the address of the HC organization. If HCs were present in multiple counties, we merged the data for the county where the largest share of HC patients lived. We included all HCs that reported serving patients in 2017 for a total analytic sample of 1,373.  **Methods, paragraph 5 “Independent variables” subsection**  The contextual control variables, extracted from AHRF, included the ratio of PCP per 5,000 individuals in the county, the percentage of individuals below the federal poverty guideline, and the percentage of minorities in the county. |
| Bias | 9 | Describe any efforts to address potential sources of bias | **Methods, paragraph 4, “Independent Variables” subsection**  The primary variable of interest was urban/rural status. HCs self-reported this status for the organization and used the same designation for all delivery sites. In some instances, HCs had several service delivery sites across both urban and rural areas, which resulted in some misclassification. We controlled for several HC organizational characteristics, patient characteristics, and contextual variables to account for any potential confounding among HC and local area factors. |
| Study size | 10 | Explain how the study size was arrived at | **Methods, paragraph 1-2 “Data and sample” subsection**  Data and sample. For this cross-sectional study, we used data from the 2017 Uniform Data System (UDS) reported by all HRSA-funded HCs on organizational characteristics and clinical quality measures. UDS is an administrative data source maintained by HRSA to monitor the Health Center Program and provide information to stakeholders. HRSA-funded HCs and other entities receiving federal funding authorized under Section 330 of the Public Health Service Act are required to report UDS data. UDS captures aggregate information at the organization level rather than individual delivery sites that operate within the organization.[19]  We merged the UDS data with the latest available relevant data from the 2016 Area Health Resource File (AHRF). AHRF is a publicly available dataset maintained by HRSA. It compiles information from over 50 sources to provide county-level data on population characteristics, health workforce availability, health care utilization, and health facilities.[22] We merged these data using the Federal Information Process Standards (FIPS) code associated with the address of the HC organization. If HCs were present in multiple counties, we merged the data for the county where the largest share of HC patients lived. We included all HCs that reported serving patients in 2017 for a total analytic sample of 1,373. |
| Quantitative variables | 11 | Explain how quantitative variables were handled in the analyses. If applicable, describe which groupings were chosen and why | **Methods, paragraph 3-5 “Dependent variables and independent variables” subsection**  **Dependent Variables**. We studied clinical quality measures that HCs are required to report. The majority of these measures were standard quality metrics and concordant with Centers for Medicare and Medicaid Services guidelines and electronically specified for automated reporting by HCs (electronic clinical quality measures).[23] These measures had national benchmarks included in the 2017 Healthcare Effectiveness Data and Information Set (HEDIS) for Medicaid Managed Care patients.[24, 25] A total of 15 measures were included in our analyses, with seven measures examining prevention-related performance, five measures associated with care management, and three measures assessing clinical outcomes. All measures were treated as continuous variables. The seven prevention measures included (1) percent of children 2 years of age immunized in accordance with recommended guidelines from the Advisory Committee on Immunization Practices, (2) patients with Pap tests as recommended (percentage of women ages 21-64 years who had cervical cytology performed every three years, and women ages 30-64 who had cervical cytology/human papillomavirus co-testing performed every five years), (3) patients ages 50–75 years old who received appropriate colorectal cancer screening, (4) adult patients 18 years and older screened for tobacco use and cessation counseling and intervention, (5) patients 12 years or older who were routinely screened for depression and received a follow-up plan, (6) adolescents and children ages 3-17 years old with documented body mass index (BMI) percentile and counseling on nutrition and exercise, and (7) percent of adults 18 years and older with documented BMI and follow-up plan. The five measures examining aspects of care management included: (1) patients ages 5-64 years old with persistent asthma who received pharmacological therapy, (2) patients with coronary artery disease and high lipid levels that received lipid lowering therapy, (3) patients with ischemic vascular disease who used aspirin or another antithrombotic drug, (4) patients seen for follow-up care within 90 days of initial HIV diagnosis, and (5) pregnant women who received prenatal care in their first trimester. Three measures examined outcomes of care, including (1) percent of patients with diabetes whose hemoglobin A1c level was greater than 9% at the last measurement for the year (poorly controlled), (2) patients with diagnosed hypertension whose blood pressure was below 140/90 (controlled) at last reading during the year, and (3) patients born to health center patients whose birthweight was below normal (2,500 grams).  **Independent Variables.** The primary variable of interest was urban/rural status. HCs self-reported this status for the organization and used the same designation for all delivery sites. In some instances, HCs had several service delivery sites across both urban and rural areas, which resulted in some misclassification. We controlled for several HC organizational characteristics, patient characteristics, and contextual variables to account for any potential confounding among HC and local area factors. The HC organizational characteristics controlled for included the organizational size indicated by the number of sites and number of patients seen in 2017. We further controlled for patient demographic and health characteristics including percent of patients ages 0–17 and ages 65 or older, patients who were racial/ethnic minorities, patients who communicated with the provider in a language other than English, patients with heart related disease, patients with diabetes or endocrine disease, patients with respiratory disease, patients with HIV, and prenatal care patients who delivered during the year. These variables controlled for HC case mix and challenges to care outcomes.  We next controlled for primary care and other care capacity and service availability using several indicators. These included the ratio of HC patients per each full-time equivalent (FTE) primary care provider (PCPs include physicians, nurse practitioners, and physician assistants) and ratio of FTE nurses per PCP, the ratio of mental health providers (psychiatrists, psychologists, licensed clinical social workers, other licensed mental health providers) per 5,000 patients, dental providers (dentists and hygienists) per 2,500 patients, enabling service staff (case managers, transportation, and translation staff) per 5,000 patients, and an index of number of services available in addition to medical care. We also included financial resource indicators, including per capita total revenues to measure success in generating revenues and percent of grant revenues from the Section 330 grants to measure success in fundraising. The contextual control variables, extracted from AHRF, included the ratio of PCP per 5,000 individuals in the county, the percentage of individuals below the federal poverty guideline, and the percentage of minorities in the county. |
| Statistical methods | 12 | (*a*) Describe all statistical methods, including those used to control for confounding | **Methods, paragraph 6 “Statistical Analysis” subsection**  **Statistical Analysis.** We compared the independent and control variables by urban/rural status using t-tests. We then developed generalized linear regression models with the logit link and binomial distribution to compare clinical quality measures after adjusting for HC patient and organizational characteristics and county-level contextual factors to control for confounding. We included only complete data for all analyses presented in this paper. All analyses were conducted using STATA v. 15, and we used the Margins command to report predicted probabilities for ease of interpretation. We discussed all statistically significant results with probability values of 0.05 or smaller. |
|  |  | (*b*) Describe any methods used to examine subgroups and interactions | **Methods, paragraph 6 “Statistical Analysis” subsection**  **Statistical Analysis.** We compared the independent and control variables by urban/rural status using t-tests. We then developed generalized linear regression models with the logit link and binomial distribution to compare clinical quality measures after adjusting for HC patient and organizational characteristics and county-level contextual factors to control for confounding. We included only complete data for all analyses presented in this paper. All analyses were conducted using STATA v. 15, and we used the Margins command to report predicted probabilities for ease of interpretation. We discussed all statistically significant results with probability values of 0.05 or smaller. |
|  |  | (*c*) Explain how missing data were addressed | **Methods, paragraph 6 “Statistical Analysis” subsection**  We included only complete data for all analyses presented in this paper. |
|  |  | (*d*) If applicable, describe analytical methods taking account of sampling strategy | Not applicable |
|  |  | (*e*) Describe any sensitivity analyses | There were no sensitivity analyses done |
| Results | | |  |
| Participants | 13* | (a) Report numbers of individuals at each stage of study—eg numbers potentially eligible, examined for eligibility, confirmed eligible, included in the study, completing follow-up, and analysed | **Methods, paragraph 2 “Data and sample” subsection**  We included all HCs that reported serving patients in 2017 for a total analytic sample of 1,373.  **Results, paragraph 1**  Table 1 indicated about 44% of HCs were rural. |
|  |  | (b) Give reasons for non-participation at each stage | Not applicable |
|  |  | (c) Consider use of a flow diagram | Not applicable |
| Descriptive data | 14* | (a) Give characteristics of study participants (eg demographic, clinical, social) and information on exposures and potential confounders | **Results, paragraph 1**  Table 1 indicated about 44% of HCs were rural. On average, rural HCs were smaller than non-rural HCs as indicated by fewer sites (7.4 [SD 8.1] vs. 8.6 [SD 9.6]) and patients (14,673 [SD 18,068] vs. 23,861[SD 26,623]). Rural HCs had more older patients (13.2% [SD 6.6%]vs 7.2% [SD 4.2%]), fewer racial/ethnic minorities (38.2% [SD 31.1%] vs 70.7% [SD 23.5%]), fewer patients speaking in non-English languages (12.5% [SD 22.2] vs 24.3% [SD 22.0%]), and less Medicaid patients (35.1% [SD 17.1%] vs. 50.7% [SD 18.6%]) than urban HCs. Rural HCs also had more patients with heart and respiratory diseases but fewer patients with HIV or prenatal patients who delivered than urban HCs. Rural HCs also differed from urban HCs in capacity of with a higher ratio of dental providers per 2,500 patients (0.8 [SD 0.8] vs 0.7 [SD 0.7]), and nurses to PCPs (0.9 [SD 0.6] vs. 0.7 [SD 0.5]), but lower PCP panel size (1,092 [SD 514] vs. 1,168 [SD 501]), ratio of mental health providers to 5,000 patients (1.6 [SD 2.1] vs. 2.2 [SD 3.9]), enabling service staff to 5,000 patients (4.5 [SD 5.6] vs. 6.0 [SD 8.5]), and offered fewer types of services (3.3 [SD 1.6] vs 3.6 [SD 1.6]). Section 330 grants represented a higher percentage of total revenue in rural HCs than urban HCs (34.0% [SD 17.9%] vs. 25.9% [SD 17.9%]). Rural HCs also had lower PCP capacity in the county overall and less racial/ethnic diversity but more patients living in poverty than urban HCs.  **Results, table 1** |
|  |  | (b) Indicate number of participants with missing data for each variable of interest | **Methods, paragraph 6 “Statistical Analysis” subsection**  We included only complete data for all analyses presented in this paper. |
| Outcome data | 15* | Report numbers of outcome events or summary measures | **Results, paragraph 2**  Unadjusted clinical quality measures showed multiple differences between rural and urban HCs including lower rates of recommended Pap tests (47% [SD 17%] vs. 53% [SD 18%]), up-to-date child immunizations (30% [SD 23%] vs 38% [SD 23%]), and body mass index percentile documentation and counseling of children and adolescents (55% [SD 26%] vs. 62% [SD 26%]) (Table 2). Among care management measures, rural HCs had lower rates of appropriate pharmacological therapy for patients with persistent asthma (83% [SD 17%] vs. 86% [SD 13%]), lipid lowering therapy for patients with coronary artery disease (78% [SD 15%] vs. 80% [SD 13%]), use of antithrombotic drugs for patients with ischemic vascular disease (76% [SD 16%] vs. 78% [SD 14%]), and linkage to care for newly diagnosed HIV patients (71% [SD 40%] vs. 84% [SD 27%]) but higher rates of early prenatal care for pregnant patients (81% [SD 16%] vs. 74% [SD 15%]) compared to urban HCs. Rural HCs had different performance rates on outcome quality measures, with 32% (SD 12%) of rural HCs reporting patients with diabetes had uncontrolled hemoglobin A1c levels (vs. 35% [SD 12%] of urban HCs) and 63% reporting patients with hypertension had their blood pressure controlled (vs. 61% of urban HCs).  **Results, table 2** |
| Main results | 16 | (*a*) Give unadjusted estimates and, if applicable, confounder-adjusted estimates and their precision (eg, 95% confidence interval). Make clear which confounders were adjusted for and why they were included | **Results, paragraphs 2-3**  Unadjusted clinical quality measures showed multiple differences between rural and urban HCs including lower rates of recommended Pap tests (47% [SD 17%] vs. 53% [SD 18%]), up-to-date child immunizations (30% [SD 23%] vs 38% [SD 23%]), and body mass index percentile documentation and counseling of children and adolescents (55% [SD 26%] vs. 62% [SD 26%]) (Table 2). Among care management measures, rural HCs had lower rates of appropriate pharmacological therapy for patients with persistent asthma (83% [SD 17%] vs. 86% [SD 13%]), lipid lowering therapy for patients with coronary artery disease (78% [SD 15%] vs. 80% [SD 13%]), use of antithrombotic drugs for patients with ischemic vascular disease (76% [SD 16%] vs. 78% [SD 14%]), and linkage to care for newly diagnosed HIV patients (71% [SD 40%] vs. 84% [SD 27%]) but higher rates of early prenatal care for pregnant patients (81% [SD 16%] vs. 74% [SD 15%]) compared to urban HCs. Rural HCs had different performance rates on outcome quality measures, with 32% (SD 12%) of rural HCs reporting patients with diabetes had uncontrolled hemoglobin A1c levels (vs. 35% [SD 12%] of urban HCs) and 63% reporting patients with hypertension had their blood pressure controlled (vs. 61% of urban HCs).  After adjusting for patient, organizational, and county-level characteristics, the differences in clinical quality measures remained statistically significant only in care management of newly diagnosed HIV patients. Rural HCs had a predicted probability of 75% [95% CI: 69%, 80%] of newly diagnosed HIV patients being linked to care in 90 days compared to 83% [95% CI: 80%, 86%] in urban HCs (Table 3). However, differences in all other clinical quality measures were explained by underlying differences in patient demographics and health status, organizational characteristics, and contextual factors to varying degrees and depending on the performance measure. For example, the difference between urban and rural HC performance on childhood immunization status was explained by the higher number of non-English speaking patients, higher rate of children at the HC, and lower rate of patients with respiratory diseases (S1 Table).  **Results, table 2-3**  **Methods, paragraph 6 “Statistical Analysis” subsection**  We then developed generalized linear regression models with the logit link and binomial distribution to compare clinical quality measures after adjusting for HC patient and organizational characteristics and county-level contextual factors to control for confounding. |
|  |  | (*b*) Report category boundaries when continuous variables were categorized | Not applicable |
|  |  | (*c*) If relevant, consider translating estimates of relative risk into absolute risk for a meaningful time period | Not applicable |
| Other analyses | 17 | Report other analyses done—eg analyses of subgroups and interactions, and sensitivity analyses | Not applicable |
| Discussion | | |  |
| Key results | 18 | Summarise key results with reference to study objectives | **Discussion, paragraph 1**  Our findings show that rural HCs had significant differences in patient, organizational, and contextual characteristics compared with urban HCs. Rural HCs also had lower performance on preventive and care management measures but better performance on outcome measures than urban HCs, though these differences were small. We found that nearly all urban-rural clinical quality measure differences could be attributed to patient, organizational, and contextual differences, with varying characteristics as the explanatory factors for performance differences on specific clinical quality measures. |
| Limitations | 19 | Discuss limitations of the study, taking into account sources of potential bias or imprecision. Discuss both direction and magnitude of any potential bias | **Discussion, paragraph 6**  Our study had limitations including a single urban or rural designation for HC organizations even if some delivery sites may not be in rural areas. However, we used delivery site addresses to determine that 11% of sites among HCs that self-designated as rural may be urban and 13% of sites among HCs that self-designated as urban may be rural. This potential discrepancy is likely to be a consequence of variations in definitions of rural designation and the lack of UDS data on individual HC sites, which requires HCs to make an overall determination even if there is an urban and rural mix among the organization’s sites. Given that our assessment found that potential misclassification is fairly uniform (11 and 13%), the bias that results is likely to weaken the associations between our outcomes of interest and urban and rural status. Additionally, because UDS data lacks information on individual HC sites, there is a potential masking of differences at site-level or patient-level. Our study is cross-sectional in nature and causal relationships between our independent and dependent variables cannot be readily determined. Furthermore, it is possible that performance among clinical measures are independently correlated and are overestimated. Our national benchmarks are based on performance measures for Medicaid managed care organizations, which are a subset of HC patients and may limit national generalizability. However, because the majority of HC patients are Medicaid beneficiaries, these national benchmarks are likely to be the most relevant. Despite this limitation, both rural and urban HCs performed well in several preventive and outcome measures. Future research should include several years of data to assess the role HC characteristics have in eliminating differences among urban and rural clinical performance over time. |
| Interpretation | 20 | Give a cautious overall interpretation of results considering objectives, limitations, multiplicity of analyses, results from similar studies, and other relevant evidence | **Discussion paragraph 7, “Policy Implications” subsection**  Policy Implications. The number of rural populations has decreased over time and their demographics have shifted.[1] Our findings highlighted comparable clinical performance between urban and rural HCs, even with the cited challenges of providing care in rural geographies. These findings stress the integral role of rural HCs in alleviating disparities in quality of care and the potential negative impact of any reductions in resources to these crucial safety net providers in rural areas. Urban/rural disparities in HIV screening and follow-up requires further attention by assessing availability of trained providers in rural areas to treat persons with HIV, identifying procedures that improve confidentiality, or providing community health education to better inform the resident and provider communities about HIV, its epidemiology, and its implication for care and treatment.[38, 42] Improving availability of providers trained in HIV care in rural areas can be achieved by federal policies that are being implemented to improve access to care in rural areas with programs leveraging HCs to diagnose, treat, prevent, and respond to HIV in communities with substantial HIV burden.[42] Other programs including loan repayment programs under the National Health Service Corps for providers working in shortage areas, state-based loan repayment programs, and the Teaching Health Center Graduate Medical Education Program, which allow HCs to operate medical residency training programs, help address emerging public health priorities.[16]  Other research indicates that lower clinical performance of HCs is linked to geographic disparities that could be alleviated by increasing the availability of resources and technical assistance.[44] HRSA has supported HC infrastructure development and provided funding to bolster the ability of these organizations to improve quality of care for low-income and uninsured patients.[15, 44] The Federal Office of Rural Health Policy has implemented programs to address access to quality health care and health professional capacity impacting rural communities. In addition, HRSA support of Health Center Controlled Networks and Primary Care Associations also provide technical resources to improve quality of care in rural and urban HCs.[44, 46]  Our findings provide support for the continuation of these programs and the identification and implementation of new programs that address performance gaps among rural HCs. Promoting quality of care among rural and urban HCs could be achieved by providing technical assistance to develop skills and resources to conduct quality improvement activities.[46] |
| Generalisability | 21 | Discuss the generalisability (external validity) of the study results | **Discussion, paragraph 6**  Our national benchmarks are based on performance measures for Medicaid managed care organizations, which are a subset of HC patients and may limit national generalizability. However, because the majority of HC patients are Medicaid beneficiaries, these national benchmarks are likely to be the most relevant. Despite this limitation, both rural and urban HCs performed well in several preventive and outcome measures. |
| Other information | | |  |
| Funding | 22 | Give the source of funding and the role of the funders for the present study and, if applicable, for the original study on which the present article is based | **Supporting information**  This research was funded by the U.S. Department of Health and Human Services (HHS), Health Resources and Services Administration (HRSA) under HRSA Contract number HHSH250201300023I (NP). |

*Give information separately for exposed and unexposed groups.

**Note:** An Explanation and Elaboration article discusses each checklist item and gives methodological background and published examples of transparent reporting. The STROBE checklist is best used in conjunction with this article (freely available on the Web sites of PLoS Medicine at http://www.plosmedicine.org/, Annals of Internal Medicine at http://www.annals.org/, and Epidemiology at http://www.epidem.com/). Information on the STROBE Initiative is available at www.strobe-statement.org.
