# Supplementary material for: Assessing clinical quality performance and staffing capacity differences between urban and rural Health Resources and Services Administration-funded health centers in the United States: A cross sectional study
Source: PLoS One. 2020 Dec 8;15(12):e0242844. doi: 10.1371/journal.pone.0242844 (PMC7723285; doi:10.1371/journal.pone.0242844)
Supplement: S1 Table — (DOCX) [file pone.0242844.s003.docx]

| **S1 Table. Performance Measures and Full Clinical Quality Performance Measure Definition** | | |
| --- | --- | --- |
| **Performance Measure** | **Abbreviated Name** | **Clinical Quality Performance Measure Definition** |
| ***Prevention*** |  |  |
| Childhood Immunization Completion | Childhood Immunization | Proportion of children 2 years of age who received age appropriate vaccines in accordance with recommended guidelines from the Advisory Committee on Immunization Practices by their 2nd birthday. |
| Receipt of Recommended Cervical Cancer Screening | Cervical Cancer Screening | Proportion of patients who received Pap tests as recommended: the proportion of women ages 21-64 years who had cervical cytology performed every three years, and women ages 30-64 who had cervical cytology/human papillomavirus co-testing performed every five years. |
| Receipt of Colorectal Cancer Screening | Colorectal Cancer Screening | Proportion of patients 50 through 75 years of age who had appropriate screening for colorectal cancer. |
| Tobacco Use and Cessation Counseling and Intervention | Tobacco Use Counseling | Proportion of patients aged 18 years of age and older who (1) were screened for tobacco use one or more times within 24 months, and if identified to be a tobacco user, (2) received cessation counseling intervention. |
| Depression Screening and Receipt of a Follow-Up Plan | Depression Screening and Follow-Up | Proportion of patients 12 years of age and older who were (1) screened for depression with a standardized tool and, if screening was positive, (2) had a follow-up plan documented. |
| Weight Assessment and Counseling for Nutrition and Physical Activity for Children and Adolescents | Child Weight Counseling | Proportion of patients 3-17 years of age with a BMI percentile, and counseling on nutrition and physical activity documented. |
| Body Mass Index (BMI) Screening and Follow-Up Plan for Adults | Adult BMI Screening | Proportion of patients 18 years of age and older with (1) BMI documented and (2) follow-up plan documented if BMI is outside normal parameters. |
| ***Care Management*** |  |  |
| Patients with Asthma Receiving Appropriate Medications | Asthma Treatment | Proportion of patients 5 through 64 years of age identified as having persistent asthma and were appropriately ordered medication. |
| Patients with Coronary Artery Diseases That Were Prescribed Lipid-Lowering Therapy | Lipid Therapy | Proportion of patients 18 years of age and older with a diagnosis of CAD who were prescribed a lipid-lowering therapy. |
| Patients with Ischemic Vascular Disease Who Used Aspirin or Another Antithrombotic Drug | Aspirin Therapy | Proportion of patients 18 years of age and older with a diagnosis of Ischemic Vascular Disease or acute myocardial infarction, coronary artery bypass graft, or percutaneous coronary interventions procedure with aspirin or another antiplatelet. |
| Patient Seen for Follow-Up Care within 90 Days of initial HIV Diagnosis | HIV Linkage to Care | Proportion of patients whose first ever HIV diagnosis was made by health center staff between October 1, of the prior year and September 30, of the measurement year and who were seen for follow-up treatment within 90 days of that first-ever diagnosis. |
| Pregnant Women Who Received Early Prenatal Care | Early Prenatal Care | Proportion of pregnant women who received prenatal care in their first trimester. |
| ***Outcomes*** |  |  |
| Patients with Diabetes with Hemoglobin A1c Greater Than 9% | Uncontrolled Diabetes | Proportion of patients 18–75 years of age with diabetes who had hemoglobin A1c (HbA1c) greater than 9.0% during the measurement period. |
| Patients with Hypertension with Blood Pressure below 140/90 | Hypertension Control | Proportion of patients 18–85 years of age who had a diagnosis of hypertension and whose blood pressure (BP) was adequately controlled (less than 140/90 mmHg) during the measurement period. |
| Patients Born Whose Birthweight Was Below Normal | Low Birth Weight | Proportion of babies of health center prenatal care patients born whose birth weight was below normal (less than 2,500 grams). |
