# Supplementary material for: Assessing clinical quality performance and staffing capacity differences between urban and rural Health Resources and Services Administration-funded health centers in the United States: A cross sectional study
Source: PLoS One. 2020 Dec 8;15(12):e0242844. doi: 10.1371/journal.pone.0242844 (PMC7723285; doi:10.1371/journal.pone.0242844)
Supplement: S2 Table — (DOCX) [file pone.0242844.s004.docx]

| **S2 Table. Unadjusted and Adjusted Predicted Probabilities of Health Center Quality Indictors by Urban and Rural Status** | | | | | | | | |
| --- | --- | --- | --- | --- | --- | --- | --- | --- |
|  | **Unadjusted^1^** | |  | **Adjusted^2^** | | | |  |
| **Measure Definition** | **Urban** | **Rural** | **p-value (Urban vs. rural)** | **Urban** | | **Rural** | | **p-value (Urban vs. rural)** |
| **Sample Size**  *n (%)* |  |  |  | 765 (56%) | | 608 (44%) | |  |
|  | *Mean (SD)* | *Mean (SD)* |  | *Predicted probability* | *95% CI* | *Predicted probability* | *95% CI* |  |
| **Prevention** |  |  |  |  |  |  |  |  |
| Childhood Immunization | 38% (23%)*** | 30% (23%) | 0.000 | 35% | [33%, 38%] | 33% | [31%, 36%] | .296 |
| Cervical Cancer Screening | 53% (18%)*** | 47% (17%) | 0.000 | 51% | [49%, 52%] | 51% | [49%, 52%] | .835 |
| Colorectal Cancer Screening | 38% (18%) | 39% (18%) | 0.269 | 39% | [37%, 40%] | 39% | [37%, 41%] | .805 |
| Tobacco Use Counseling | 86% (13%) | 85% (16%) | 0.316 | 86% | [85%, 87%] | 86% | [84%, 87%] | .834 |
| Depression Screening and Follow-Up | 64% (23%) | 64% (25%) | 0.854 | 64% | [62%, 66%] | 66% | [63%, 68%] | .276 |
| Child Weight Counseling | 62% (26%)*** | 55% (26%) | 0.000 | 60% | [58%, 62%] | 59% | [56%, 61%] | .568 |
| Adult BMI Screening | 62% (21%) | 61% (23%) | 0.449 | 62% | [60%, 64%] | 62% | [60%, 64%] | .847 |
| **Care Management** |  |  |  |  |  |  |  |  |
| Asthma Treatment | 86% (13%)** | 83% (17%) | 0.002 | 85% | [84%, 86%] | 86% | [84%, 87%] | .615 |
| Lipid Therapy | 80% (13%)** | 78% (15%) | 0.001 | 80% | [79%, 81%] | 79% | [78%, 80%] | .518 |
| Aspirin Therapy | 78% (14%)** | 76% (16%) | 0.006 | 78% | [77%, 80%] | 77% | [75%, 78%] | .111 |
| HIV Linkage to Care | 84% (27%)*** | 71% (40%) | 0.000 | 83%* | [80%, 86%] | 75% | [69%, 80%] | .011 |
| Early Prenatal Care | 74% (15%)*** | 81% (16%) | 0.000 | 77% | [76%, 78%] | 77% | [76%, 79%] | .952 |
| **Outcomes** |  |  |  |  |  |  |  |  |
| Uncontrolled Diabetes | 35% (12%)*** | 32% (12%) | 0.000 | 33% | [32%, 34%] | 34% | [33%, 35%] | .723 |
| Hypertension Control | 61% (10%)** | 63% (11%) | 0.006 | 62% | [61%, 63%] | 62% | [61%, 63%] | .829 |
| Low Birth Weight | 10% (12%) | 10% (14%) | 0.677 | 11% | [9%, 12%] | 9% | [8%, 11%] | .225 |
| Notes: ^1^ Analyses involved comparing urban and rural health center status using t-tests. | | | | | | | | |
| ^2^ Adjusted analyses were conducted using fractional outcome regression models using the logit distribution. | | | | | | | | |
| Standard deviation in parentheses. | | | | | | | | |
| BMI, body mass index; CAD, coronary artery disease; IVD, ischemic vascular disease; HIV, human immunodeficiency virus; HbA1c, Hemoglobin A1c ; SD, standard deviation; HC, health center; HEDIS, Healthcare Effectiveness Data and Information Set; CI, confidence interval. | | | | | | | | |
| Statistically significant at *p<0.05; **p<0.01; ***p<0.001 comparing urban vs. rural. | | | | | | | | |
