# Supplementary material for: Assessing clinical quality performance and staffing capacity differences between urban and rural Health Resources and Services Administration-funded health centers in the United States: A cross sectional study
Source: PLoS One. 2020 Dec 8;15(12):e0242844. doi: 10.1371/journal.pone.0242844 (PMC7723285; doi:10.1371/journal.pone.0242844)
Supplement: S3 Table — (DOCX) [file pone.0242844.s005.docx]

| **S3 Table. Regression Models of Prevention Quality Performance Indicators** | | | | | | | | | | | | | | |
| --- | --- | --- | --- | --- | --- | --- | --- | --- | --- | --- | --- | --- | --- | --- |
|  | Up-to-Date Childhood Immunization Completion | | Receipt of Recommended Cervical Cancer Screening | | Receipt of Colorectal Cancer Screening | | Tobacco Use and Cessation Counseling and Intervention | | Depression Screening and Receipt of a Follow-Up Plan | | Weight Assessment and Counseling for Nutrition and Physical Activity for Children and Adolescents | | Body Mass Index (BMI) Screening and Follow-Up Plan for Adults | |
| Sample size | 1,203 | | 1,233 | | 1,231 | | 1,233 | | 1,233 | | 1,225 | | 1,233 | |
|  | OR | 95% CI | OR | 95% CI | OR | 95% CI | OR | 95% CI | OR | 95% CI | OR | 95% CI | OR | 95% CI |
| ***Urban*** | 1.1 | [0.92,1.30] | 0.99 | [0.89,1.10] | 0.99 | [0.88,1.11] | 1.02 | [0.86,1.20] | 0.91 | [0.77,1.08] | 1.05 | [0.88,1.25] | 0.99 | [0.85,1.14] |
| ***Organization Size*** |  |  |  |  |  |  |  |  |  |  |  |  |  |  |
| Average number of sites | 1 | [1.00,1.01] | 1 | [0.99,1.00] | 1 | [0.99,1.00] | 1 | [1.00,1.01] | 1 | [0.99,1.01] | 1.01* | [1.00,1.02] | 1.01 | [1.00,1.01] |
| Average number of patients seen during the year | 1.01 | [0.98,1.04] | 1.03** | [1.01,1.05] | 1.03** | [1.01,1.05] | 1.03 | [1.00,1.07] | 1.04* | [1.01,1.09] | 1 | [0.97,1.04] | 1 | [0.97,1.04] |
| ***Patient Characteristics*** |  |  |  |  |  |  |  |  |  |  |  |  |  |  |
| Percent of patients that were racial/ethnic minorities | 0.95 | [0.58,1.56] | 0.86 | [0.65,1.13] | 0.60** | [0.44,0.82] | 0.43*** | [0.28,0.66] | 0.83 | [0.56,1.24] | 0.87 | [0.56,1.37] | 0.7 | [0.48,1.03] |
| Percent of patients that spoke with primary care provider (PCP) in a language other than English | 1.89** | [1.25,2.87] | 1.98*** | [1.55,2.54] | 2.40*** | [1.82,3.16] | 3.11*** | [2.05,4.74] | 1.11 | [0.77,1.61] | 1.63* | [1.08,2.45] | 1.41 | [1.00,2.00] |
| Percent of patients 65 years and older | 2.75 | [0.39,19.26] | 1.05 | [0.37,2.98] | 5.25** | [1.60,17.19] | 4.83 | [1.00,23.45] | 0.27 | [0.05,1.37] | 0.63 | [0.11,3.60] | 0.68 | [0.17,2.79] |
| Percent of patients between 0--17 years | 2.73** | [1.35,5.52] | 1.71* | [1.12,2.61] | 1.86* | [1.16,3.00] | 1.42 | [0.71,2.85] | 1.5 | [0.78,2.88] | 3.69*** | [1.87,7.30] | 2.13** | [1.24,3.65] |
| Percent of patients with heart related disease | 85.27 | [0.36,20471.96] | 25.81 | [0.82,809.14] | 667.86*** | [14.04,31764.57] | 0.12 | [0.00,51.26] | 0.14 | [0.00,31.60] | 12.99 | [0.05,3352.53] | 1.58 | [0.02,126.67] |
| Percent of patients with diabetes or endocrine diseases | 8.49 | [0.74,97.93] | 5.94* | [1.42,24.89] | 16.89*** | [3.39,84.16] | 17.90* | [1.51,212.62] | 55.23*** | [5.69,536.37] | 67.91*** | [6.88,670.51] | 33.89*** | [4.63,248.04] |
| Percent of patients with respiratory diseases | 0.00*** | [0.00,0.03] | 0.01*** | [0.00,0.17] | 0.00*** | [0.00,0.07] | 0.07 | [0.00,4.49] | 4.82 | [0.07,314.78] | 0.17 | [0.00,14.51] | 5.86 | [0.16,208.94] |
| Percent of patients with HIV | 2.19 | [0.07,69.77] | 2.47 | [0.51,11.99] | 0.49 | [0.08,3.08] | 4.42 | [0.55,35.35] | 1.16 | [0.11,12.61] | 1.65 | [0.06,48.54] | 30.62*** | [4.89,191.71] |
| Percent of prenatal care patients who delivered during the year | 167.88 | [0.39,71682.87] | 2.56e+08*** | [3.58e+06,1.82e+10] | 271.54* | [3.69,19966.51] | 381.4 | [0.01,1.34e+07] | 281.66 | [0.24,334223.60] | 6231.82* | [3.44,1.13e+07] | 1.2 | [0.00,517.78] |
| Percent of Medicaid Patients | 1.02 | [0.64,1.64] | 0.84 | [0.64,1.10] | 1.14 | [0.84,1.54] | 1.57 | [1.00,2.47] | 0.88 | [0.58,1.35] | 1.15 | [0.73,1.81] | 1.13 | [0.77,1.64] |
| ***PCP Staffing and Capacity*** |  |  |  |  |  |  |  |  |  |  |  |  |  |  |
| PCP Panel Size (Patients Per Provider) | 1 | [1.00,1.00] | 1 | [1.00,1.00] | 1 | [1.00,1.00] | 1 | [1.00,1.00] | 1.00* | [1.00,1.00] | 1.00* | [1.00,1.00] | 1.00** | [1.00,1.00] |
| Ratio of nurses to PCP | 1.08 | [0.96,1.21] | 1.04 | [0.96,1.12] | 1.04 | [0.96,1.12] | 1 | [0.89,1.13] | 1 | [0.90,1.12] | 1.05 | [0.93,1.19] | 0.95 | [0.86,1.06] |
| ***Additional Staffing and Capacity*** |  |  |  |  |  |  |  |  |  |  |  |  |  |  |
| Ratio of mental health provider per 5,000 patients | 1.01 | [0.99,1.03] | 1 | [0.98,1.02] | 0.99 | [0.97,1.01] | 1 | [0.98,1.03] | 1.03** | [1.01,1.05] | 1.01 | [0.98,1.04] | 1.02 | [1.00,1.04] |
| Ratio of dental provider per 2,500 patients | 0.94 | [0.85,1.04] | 1.09** | [1.03,1.15] | 1.13*** | [1.07,1.20] | 1.08 | [0.99,1.19] | 1.14** | [1.04,1.24] | 1.01 | [0.91,1.13] | 1.07 | [0.99,1.16] |
| Ratio of enabling service staff per 5,000 patients | 0.99 | [0.98,1.01] | 0.99 | [0.99,1.00] | 0.99 | [0.99,1.00] | 0.99 | [0.98,1.00] | 1 | [0.99,1.01] | 1.01 | [0.99,1.02] | 1 | [0.99,1.02] |
| Average number of services provided in addition to medical care | 1 | [0.96,1.04] | 0.97* | [0.95,1.00] | 0.98 | [0.96,1.01] | 1.01 | [0.96,1.05] | 0.95* | [0.90,0.99] | 1.02 | [0.97,1.07] | 1.03 | [0.99,1.07] |
| ***Financial Resources*** |  |  |  |  |  |  |  |  |  |  |  |  |  |  |
| Per capita total revenues | 1.06 | [0.93,1.21] | 0.97 | [0.91,1.04] | 1.11** | [1.03,1.20] | 0.97 | [0.90,1.03] | 0.89* | [0.81,0.98] | 0.86 | [0.73,1.01] | 0.84*** | [0.76,0.92] |
| Proportion of total revenues that are from 330 grants | 0.91 | [0.50,1.64] | 0.49*** | [0.35,0.68] | 0.55** | [0.38,0.81] | 1.18 | [0.72,1.95] | 1.1 | [0.68,1.78] | 0.93 | [0.55,1.58] | 1.14 | [0.74,1.76] |
| ***Contextual Characteristics*** |  |  |  |  |  |  |  |  |  |  |  |  |  |  |
| Ratio of PCP per 5,000 population in county | 1.07*** | [1.03,1.10] | 1.05*** | [1.02,1.07] | 1.04** | [1.01,1.06] | 1.04 | [1.00,1.08] | 1.01 | [0.97,1.05] | 1.02 | [0.97,1.06] | 0.98 | [0.95,1.01] |
| Proportion below federal poverty guideline in county | 0.99 | [0.98,1.01] | 1 | [0.99,1.01] | 1.01 | [1.00,1.01] | 1 | [0.99,1.01] | 1 | [0.99,1.01] | 1.01 | [0.99,1.02] | 1.01 | [1.00,1.02] |
| Proportion of minority in county | 0.97 | [0.61,1.55] | 1.07 | [0.81,1.42] | 0.93 | [0.68,1.28] | 1.01 | [0.63,1.61] | 1.11 | [0.72,1.72] | 1.17 | [0.73,1.87] | 1.46 | [0.97,2.21] |
| Analyses were conducted using fractional outcome regression models using the logit distribution. | | | | | | | | | | | | | | |
| Statistically significant at *p<0.05; **p<0.01; ***p<0.001. | | | | | | | | | | | | | | |
| BMI, body mass index; CAD, coronary artery disease; IVD, ischemic vascular disease; HIV, human immunodeficiency virus; HbA1c, Hemoglobin A1c; Coef., beta coefficient; CI, confidence interval. | | | | | | | | | | | | | | |
