# Supplementary material for: Assessing clinical quality performance and staffing capacity differences between urban and rural Health Resources and Services Administration-funded health centers in the United States: A cross sectional study
Source: PLoS One. 2020 Dec 8;15(12):e0242844. doi: 10.1371/journal.pone.0242844 (PMC7723285; doi:10.1371/journal.pone.0242844)
Supplement: S4 Table — (DOCX) [file pone.0242844.s006.docx]

| **S4 Table. Regression Models of Care Management Quality Indicators** | | | | | | | | | | |
| --- | --- | --- | --- | --- | --- | --- | --- | --- | --- | --- |
|  | Patients with Asthma Receiving Appropriate Medications | | Patients with Coronary Artery Diseases That Were Prescribed Lipid-Lowering Therapy | | Patients with Ischemic Vascular Disease Who Used Aspirin or Another Antithrombotic Drug | | Patient Seen for Follow-Up Care within 90 Days of initial HIV Diagnosis | | Pregnant Women Who Received Early Prenatal Care | |
| Sample size | 1,226 | | 1,228 | | 1,230 | | 712 | | 1,178 | |
|  | OR | 95% CI | OR | 95% CI | OR | 95% CI | OR | 95% CI | OR | 95% CI |
| ***Urban*** | 0.95 | [0.78,1.15] | 1.04 | [0.92,1.17] | 1.12 | [0.98,1.27] | 1.68* | [1.13,2.52] | 1 | [0.86,1.15] |
| ***Organization Size*** |  |  |  |  |  |  |  |  |  |  |
| Average number of sites | 1 | [0.99,1.00] | 1 | [1.00,1.01] | 1 | [0.99,1.01] | 1.01 | [0.99,1.03] | 0.99** | [0.99,1.00] |
| Average number of patients seen during the year | 1.03 | [0.99,1.08] | 0.98 | [0.96,1.01] | 1.02 | [0.99,1.05] | 1.02 | [0.94,1.11] | 1.03* | [1.01,1.05] |
| ***Patient Characteristics*** |  |  |  |  |  |  |  |  |  |  |
| Percent of patients that were racial/ethnic minorities | 0.78 | [0.48,1.26] | 0.86 | [0.58,1.25] | 0.66* | [0.46,0.94] | 2.32 | [0.73,7.37] | 0.46*** | [0.32,0.65] |
| Percent of patients that spoke with primary care provider in a language other than English | 2.07*** | [1.38,3.12] | 1.61** | [1.15,2.25] | 2.01*** | [1.42,2.84] | 3.52* | [1.28,9.72] | 1.52** | [1.14,2.02] |
| Percent of patients of patients 65 years and older | 0.65 | [0.09,4.65] | 0.44 | [0.12,1.60] | 0.78 | [0.22,2.76] | 0.07 | [0.00,6.04] | 58.46*** | [11.21,304.89] |
| Percent of patients between 0--17 years | 2.02 | [0.99,4.13] | 1.51 | [0.88,2.61] | 1.24 | [0.72,2.13] | 1.16 | [0.24,5.70] | 1.25 | [0.71,2.18] |
| Percent of patients with heart related disease | 3.59 | [0.01,1585.99] | 2.63 | [0.04,188.56] | 10.17 | [0.16,628.47] | 4.7 | [0.00,4.44e+06] | 0.02 | [0.00,3.57] |
| Percent of patients with diabetes or endocrine diseases | 6.29 | [0.60,65.68] | 13.59** | [2.00,92.15] | 3.23 | [0.55,18.86] | 0.02 | [0.00,3.83] | 0.35 | [0.06,1.95] |
| Percent of patients with respiratory diseases | 11.97 | [0.08,1799.51] | 2.53 | [0.07,89.80] | 4.9 | [0.16,148.36] | 3047.13 | [0.01,8.32e+08] | 0.12 | [0.00,6.34] |
| Percent of patients with HIV | 2.33 | [0.21,26.49] | 38.64*** | [4.92,303.62] | 2.91 | [0.47,18.02] | 4842.35* | [2.66,8.83e+06] | 0.18 | [0.00,9.67] |
| Percent of prenatal care patients who delivered during the year | 747.16 | [0.24,2.32e+06] | 316.12* | [1.66,60157.80] | 49.39 | [0.20,12496.02] | 0 | [0.00,10.10] | 0.00*** | [0.00,0.00] |
| Percent of Medicaid patients | 1.07 | [0.66,1.74] | 0.89 | [0.64,1.24] | 0.81 | [0.56,1.17] | 0.91 | [0.31,2.64] | 1.23 | [0.87,1.74] |
| ***Primary Care Provider Staffing and Capacity*** |  |  |  |  |  |  |  |  |  |  |
| PCP Panel Size (Patients Per Provider) | 1 | [1.00,1.00] | 1.00* | [1.00,1.00] | 1 | [1.00,1.00] | 1 | [1.00,1.00] | 1 | [1.00,1.00] |
| *Ratio of nurses to PCP* | 0.96 | [0.82,1.12] | 1.06 | [0.97,1.17] | 1.13* | [1.02,1.24] | 1.29 | [0.94,1.75] | 0.89* | [0.80,0.98] |
| ***Additional Staffing and Capacity*** |  |  |  |  |  |  |  |  |  |  |
| Ratio of mental health provider per 5,000 patients | 1.01 | [0.98,1.04] | 1.01 | [0.98,1.04] | 1 | [0.98,1.03] | 0.99 | [0.94,1.05] | 0.98 | [0.96,1.01] |
| *Ratio of dental provider per 2,500 patients* | 1.09 | [0.98,1.23] | 1 | [0.92,1.09] | 1.03 | [0.95,1.13] | 0.95 | [0.71,1.26] | 0.92 | [0.83,1.01] |
| Ratio of enabling service staff per 5,000 patients | 1 | [0.98,1.01] | 0.99* | [0.98,1.00] | 0.99 | [0.97,1.00] | 1.02 | [0.99,1.06] | 1.01 | [1.00,1.02] |
| Average number of services provided in addition to medical care | 0.99 | [0.94,1.04] | 1.04* | [1.00,1.07] | 1.01 | [0.97,1.04] | 0.99 | [0.89,1.11] | 0.97 | [0.94,1.01] |
| ***Financial Resources*** |  |  |  |  |  |  |  |  |  |  |
| Per capita total revenues | 0.95 | [0.87,1.04] | 0.86** | [0.77,0.96] | 1 | [0.94,1.07] | 0.86 | [0.60,1.24] | 1.19* | [1.03,1.37] |
| Proportion of total revenues that are from 330 grants | 0.86 | [0.48,1.55] | 0.88 | [0.58,1.34] | 0.68 | [0.44,1.07] | 0.82 | [0.24,2.84] | 0.89 | [0.57,1.38] |
| ***Contextual characteristics*** |  |  |  |  |  |  |  |  |  |  |
| Ratio of PCP per 5,000 population in county | 1.09*** | [1.04,1.14] | 1.03 | [0.99,1.06] | 1.04* | [1.01,1.08] | 1.04 | [0.93,1.15] | 1 | [0.97,1.04] |
| Proportion below federal poverty guideline in county | 0.99 | [0.98,1.01] | 1 | [0.99,1.01] | 0.99 | [0.99,1.00] | 1.01 | [0.98,1.04] | 1.01* | [1.00,1.03] |
| Proportion of minority in county | 1.36 | [0.85,2.15] | 1 | [0.69,1.46] | 1.29 | [0.90,1.85] | 0.37 | [0.11,1.22] | 0.77 | [0.54,1.11] |
| Analyses were conducted using fractional outcome regression models using the logit distribution. | | | | | | | | | | |
| Statistically significant at *p<0.05; **p<0.01; ***p<0.001. | | | | | | | | | | |
| BMI, body mass index; CAD, coronary artery disease; IVD, ischemic vascular disease; HIV, human immunodeficiency virus; HbA1c, Hemoglobin A1c; Coef., beta coefficient; CI, confidence interval. | | | | | | | | | | |
