# Supplementary material for: Assessing clinical quality performance and staffing capacity differences between urban and rural Health Resources and Services Administration-funded health centers in the United States: A cross sectional study
Source: PLoS One. 2020 Dec 8;15(12):e0242844. doi: 10.1371/journal.pone.0242844 (PMC7723285; doi:10.1371/journal.pone.0242844)
Supplement: S5 Table — (DOCX) [file pone.0242844.s007.docx]

| **S5 Table. Regression Models of Outcome Quality Indicators** | | | | | | |
| --- | --- | --- | --- | --- | --- | --- |
|  | Patients with Diabetes with Hemoglobin A1c Greater Than 9% | | Patients with Hypertension with Blood Pressure below 140/90 | | Patients Born Whose Birthweight Was Below Normal | |
| **Sample Size** | 1,233 | | 1,233 | | 1,139 | |
|  | OR | 95% CI | OR | 95% CI | OR | 95% CI |
| ***Urban*** | 0.99 | [0.92,1.06] | 0.99 | [0.93,1.06] | 1.18 | [0.90,1.54] |
| ***Organization Size*** |  |  |  |  |  |  |
| Average number of sites | 1 | [1.00,1.00] | 1 | [1.00,1.00] | 0.99* | [0.98,1.00] |
| Average number of patients seen during the year | 0.98* | [0.97,1.00] | 1.01 | [1.00,1.02] | 1.02 | [0.99,1.06] |
| ***Patient Characteristics*** |  |  |  |  |  |  |
| Percent of patients that were racial/ethnic minorities | 1.51*** | [1.22,1.87] | 0.62*** | [0.52,0.74] | 2.03* | [1.03,4.02] |
| Percent of patients that spoke with primary care provider (PCP) in a language other than English | 0.62*** | [0.53,0.73] | 1.75*** | [1.50,2.03] | 0.33*** | [0.20,0.56] |
| Percent of patients of patients 65 years and older | 0.16*** | [0.07,0.36] | 1.85 | [0.92,3.71] | 2.34 | [0.19,28.66] |
| Percent of patients between 0--17 years | 0.84 | [0.61,1.16] | 1.22 | [0.90,1.66] | 0.34* | [0.12,0.95] |
| Percent of patients with heart related disease | 0.07 | [0.00,1.12] | 4.79 | [0.39,58.31] | 0.17 | [0.00,142.23] |
| Percent of patients with diabetes or endocrine diseases | 0.24** | [0.09,0.66] | 2.96* | [1.15,7.58] | 0.51 | [0.01,39.29] |
| Percent of patients with respiratory diseases | 28.50** | [3.25,250.03] | 0.89 | [0.16,5.03] | 4.59 | [0.01,3937.40] |
| Percent of patients with HIV | 0.29** | [0.12,0.72] | 0.86 | [0.41,1.79] | 24.31 | [0.20,2931.67] |
| Percent of prenatal care patients who delivered during the year | 1.14 | [0.07,18.62] | 2.87 | [0.22,37.36] | 0.00** | [0.00,0.02] |
| Percent of Medicaid Patients | 0.91 | [0.75,1.10] | 1.47*** | [1.23,1.75] | 1.72 | [0.85,3.49] |
| ***PCP Staffing and Capacity*** |  |  |  |  |  |  |
| PCP Panel Size (Patients Per Provider) | 1 | [1.00,1.00] | 1 | [1.00,1.00] | 1 | [1.00,1.00] |
| Ratio of nurses to PCP | 0.98 | [0.93,1.03] | 1.02 | [0.97,1.06] | 1.1 | [0.94,1.28] |
| ***Additional Staffing and Capacity*** |  |  |  |  |  |  |
| Ratio of mental health provider per 5,000 patients | 1 | [0.99,1.01] | 1 | [0.99,1.01] | 1.03 | [0.99,1.08] |
| Ratio of dental provider per 2,500 patients | 0.95** | [0.91,0.98] | 1.03 | [0.99,1.06] | 0.94 | [0.82,1.07] |
| Ratio of enabling service staff per 5,000 patients | 1 | [1.00,1.00] | 1 | [0.99,1.00] | 1 | [0.97,1.02] |
| Average number of services provided in addition to medical care | 1.02 | [1.00,1.04] | 0.99 | [0.97,1.01] | 1 | [0.93,1.08] |
| ***Financial Resources*** |  |  |  |  |  |  |
| Per capita total revenues | 0.99 | [0.96,1.02] | 1.03* | [1.00,1.06] | 0.93 | [0.78,1.11] |
| Proportion of total revenues that are from 330 grants | 1.55*** | [1.22,1.96] | 0.74** | [0.60,0.92] | 1.93 | [0.76,4.92] |
| ***Contextual Characteristics*** |  |  |  |  |  |  |
| Ratio of PCP per 5,000 population in county | 0.99 | [0.97,1.00] | 0.99 | [0.98,1.01] | 0.95 | [0.90,1.01] |
| Proportion below federal poverty guideline in county | 1 | [0.99,1.00] | 1 | [0.99,1.00] | 1.01 | [1.00,1.03] |
| Proportion of minority in county | 1.23* | [1.01,1.50] | 1.01 | [0.84,1.21] | 0.48** | [0.29,0.79] |
| Analyses were conducted using fractional outcome regression models using the logit distribution. | | | | | | |
| Statistically significant at *p<0.05; **p<0.01; ***p<0.001. | | | | | | |
| BMI, body mass index; CAD, coronary artery disease; IVD, ischemic vascular disease; HIV, human immunodeficiency virus; HbA1c, Hemoglobin A1c; Coef., beta coefficient; CI, confidence interval. | | | | | | |
