# Supplementary material for: Assessing clinical quality performance and staffing capacity differences between urban and rural Health Resources and Services Administration-funded health centers in the United States: A cross sectional study
Source: PLoS One. 2020 Dec 8;15(12):e0242844. doi: 10.1371/journal.pone.0242844 (PMC7723285; doi:10.1371/journal.pone.0242844)
Supplement: S6 Table — (DOCX) [file pone.0242844.s008.docx]

| **S6 Table. Logistic Regression Models of Health Centers that Met Prevention Quality Performance Indicator Benchmarks** | | | | | | | | | | |
| --- | --- | --- | --- | --- | --- | --- | --- | --- | --- | --- |
|  | Met Up-to-Date Childhood Immunization Completion Performance Measure | | Met Receipt of Recommended Cervical Cancer Screening Performance Measure | | Met Tobacco Use and Cessation Counseling and Intervention Performance Measure | | Met Weight Assessment and Counseling for Nutrition and Physical Activity for Children and Adolescents Performance Measure | | Met Body Mass Index (BMI) Screening and Follow-Up Plan for Adults Performance Measure | |
| Sample size | 1,203 | | 1,233 | | 1,233 | | 1,225 | | 1,233 | |
|  | OR | 95% CI | OR | 95% CI | OR | 95% CI | OR | 95% CI | OR | 95% CI |
| ***Urban*** | 1.38 | [0.97,1.97] | 0.97 | [0.65,1.43] | 1.36 | [0.87,2.13] | 1.16 | [0.81,1.65] | 1.16 | [0.75,1.78] |
| ***Organization Size*** |  |  |  |  |  |  |  |  |  |  |
| Average number of sites | 1.01 | [0.99,1.03] | 0.98 | [0.96,1.01] | 1.01 | [0.99,1.04] | 1.02 | [1.00,1.04] | 1.01 | [0.98,1.03] |
| Average number of patients seen during the year | 1.05 | [0.97,1.14] | 1.06 | [0.98,1.16] | 1 | [0.89,1.13] | 0.96 | [0.89,1.04] | 1.03 | [0.94,1.13] |
| ***Patient Characteristics*** |  |  |  |  |  |  |  |  |  |  |
| Percent of patients that were racial/ethnic minorities | 0.96 | [0.39,2.41] | 0.56 | [0.20,1.54] | 0.12*** | [0.04,0.38] | 1.13 | [0.45,2.80] | 0.45 | [0.14,1.39] |
| Percent of patients that spoke with primary care provider (PCP) in a language other than English | 4.94*** | [2.13,11.46] | 7.03*** | [2.90,17.06] | 13.32*** | [4.06,43.64] | 2.80* | [1.26,6.27] | 1.5 | [0.54,4.14] |
| Percent of patients 65 years and older | 6.58 | [0.19,224.69] | 0.82 | [0.02,41.17] | 523.09** | [6.69,40928.64] | 0.43 | [0.01,14.95] | 0.09 | [0.00,7.14] |
| Percent of patients between 0--17 years | 10.83*** | [2.70,43.39] | 1.49 | [0.33,6.76] | 1.58 | [0.31,8.05] | 7.01** | [1.83,26.87] | 3.07 | [0.60,15.61] |
| Percent of patients with heart related disease | 153539.67* | [2.89,8.17e+09] | 341357.24* | [2.75,4.24e+10] | 0 | [0.00,5.45] | 35.56 | [0.00,1.59e+06] | 643.05 | [0.00,2.10e+08] |
| Percent of patients with diabetes or endocrine diseases | 7.58 | [0.07,767.33] | 4.93 | [0.03,848.82] | 193.28 | [0.74,50720.43] | 21 | [0.23,1948.30] | 14.88 | [0.07,3232.15] |
| Percent of patients with respiratory diseases | 0.00** | [0.00,0.02] | 0.00** | [0.00,0.00] | 0.01 | [0.00,344.69] | 14.62 | [0.00,85675.59] | 672.03 | [0.03,1.47e+07] |
| Percent of patients with HIV | 26.3 | [0.08,8596.23] | 3.34 | [0.01,2121.87] | 0.17 | [0.00,26.91] | 30.28 | [0.10,9041.19] | 27.03 | [0.01,83002.03] |
| Percent of prenatal care patients who delivered during the year | 5.76e+06* | [1.60,2.07e+13] | 2.83e+23*** | [1.00e+16,8.01e+30] | 4.77E+09 | [0.89,2.56e+19] | 1.90e+07* | [7.95,4.53e+13] | 0.06 | [0.00,1.01e+07] |
| Percent of Medicaid Patients | 0.57 | [0.23,1.42] | 0.63 | [0.23,1.72] | 3.52* | [1.17,10.58] | 0.95 | [0.39,2.30] | 0.94 | [0.32,2.78] |
| ***PCP Staffing and Capacity*** |  |  |  |  |  |  |  |  |  |  |
| PCP Panel Size (Patients Per Provider) | 1 | [1.00,1.00] | 1 | [1.00,1.00] | 1 | [1.00,1.00] | 1.00* | [1.00,1.00] | 1.00* | [1.00,1.00] |
| Ratio of nurses to PCP | 1.25 | [0.97,1.61] | 1.35* | [1.03,1.77] | 1.03 | [0.76,1.40] | 1.15 | [0.90,1.47] | 0.89 | [0.66,1.21] |
| ***Additional Staffing and Capacity*** |  |  |  |  |  |  |  |  |  |  |
| Ratio of mental health provider per 5,000 patients | 1.03 | [0.98,1.08] | 0.99 | [0.92,1.06] | 0.98 | [0.93,1.03] | 1.04 | [0.99,1.09] | 1.04 | [0.97,1.11] |
| Ratio of dental provider per 2,500 patients | 1.03 | [0.84,1.26] | 1.37** | [1.10,1.71] | 1.04 | [0.82,1.32] | 1.09 | [0.89,1.33] | 1.17 | [0.91,1.50] |
| Ratio of enabling service staff per 5,000 patients | 1 | [0.97,1.03] | 1 | [0.96,1.03] | 0.98 | [0.96,1.01] | 1 | [0.98,1.03] | 1 | [0.97,1.04] |
| Average number of services provided in addition to medical care | 0.98 | [0.89,1.08] | 0.92 | [0.83,1.02] | 0.98 | [0.87,1.10] | 0.99 | [0.90,1.08] | 1.02 | [0.91,1.14] |
| ***Financial Resources*** |  |  |  |  |  |  |  |  |  |  |
| Per capita total revenues | 0.95 | [0.77,1.17] | 0.77 | [0.55,1.07] | 1.01 | [0.83,1.24] | 0.8 | [0.60,1.07] | 0.65 | [0.41,1.02] |
| Proportion of total revenues that are from 330 grants | 0.65 | [0.24,1.82] | 0.10*** | [0.03,0.33] | 1.08 | [0.34,3.40] | 1.09 | [0.40,3.02] | 2.06 | [0.62,6.86] |
| ***Contextual Characteristics*** |  |  |  |  |  |  |  |  |  |  |
| Ratio of PCP per 5,000 population in county | 1.16*** | [1.06,1.27] | 1.1 | [1.00,1.21] | 1.15* | [1.02,1.28] | 1.03 | [0.94,1.12] | 0.89 | [0.80,1.00] |
| Proportion below federal poverty guideline in county | 0.99 | [0.96,1.02] | 0.99 | [0.96,1.02] | 1.01 | [0.98,1.04] | 1.03* | [1.00,1.06] | 1.02 | [0.99,1.06] |
| Proportion of minority in county | 0.85 | [0.32,2.25] | 2.26 | [0.77,6.59] | 1.69 | [0.49,5.81] | 1.47 | [0.56,3.86] | 3.97* | [1.21,13.05] |
| Analyses were conducted using logistic regression models. | | | | | | | | | | |
| Statistically significant at *p<0.05; **p<0.01; ***p<0.001. | | | | | | | | | | |
| BMI, body mass index; CAD, coronary artery disease; IVD, ischemic vascular disease; HIV, human immunodeficiency virus; HbA1c, Hemoglobin A1c; Coef., beta coefficient; CI, confidence interval; OR, odds ratio. | | | | | | | | | | |
