# Supplementary material for: Assessing clinical quality performance and staffing capacity differences between urban and rural Health Resources and Services Administration-funded health centers in the United States: A cross sectional study
Source: PLoS One. 2020 Dec 8;15(12):e0242844. doi: 10.1371/journal.pone.0242844 (PMC7723285; doi:10.1371/journal.pone.0242844)
Supplement: S7 Table — (DOCX) [file pone.0242844.s009.docx]

| **S7 Table. Logistic Regression Models of Health Centers that Met Care Management Quality Performance Indicator Benchmarks** | | | | | | |
| --- | --- | --- | --- | --- | --- | --- |
|  | Met Patients with Asthma Receiving Appropriate Medications Performance Measure | | Met Patients with Coronary Artery Diseases That Were Prescribed Lipid-Lowering Therapy Performance Measure | | Met Pregnant Women Who Received Early Prenatal Care Performance Measure | |
| Sample size | 1,226 | | 1,228 | | 1,178 | |
|  | OR | 95% CI | OR | 95% CI | OR | 95% CI |
| ***Urban*** | 1.17 | [0.56,2.42] | 1.07 | [0.74,1.55] | 0.94 | [0.66,1.36] |
| ***Organization Size*** |  |  |  |  |  |  |
| Average number of sites | 0.97 | [0.93,1.02] | 1.01 | [0.99,1.03] | 0.97** | [0.95,0.99] |
| Average number of patients seen during the year | 1.71** | [1.18,2.50] | 0.97 | [0.89,1.05] | 1.11* | [1.02,1.20] |
| ***Patient Characteristics*** |  |  |  |  |  |  |
| Percent of patients that were racial/ethnic minorities | 0.44 | [0.07,2.63] | 1.13 | [0.44,2.91] | 0.27** | [0.10,0.70] |
| Percent of patients that spoke with primary care provider in a language other than English | 3.16 | [0.49,20.31] | 2.90* | [1.18,7.12] | 3.44** | [1.44,8.21] |
| Percent of patients of patients 65 years and older | 2.37 | [0.00,1238.40] | 0.06 | [0.00,1.83] | 3411.46*** | [74.16,156930.22] |
| Percent of patients between 0--17 years | 8.37 | [0.60,117.69] | 2.38 | [0.57,9.90] | 1.26 | [0.30,5.21] |
| Percent of patients with heart related disease | 1.29E+07 | [0.00,1.39e+17] | 0.28 | [0.00,15883.27] | 0 | [0.00,124.68] |
| Percent of patients with diabetes or endocrine diseases | 163.3 | [0.03,858265.19] | 1805.36** | [13.70,237908.15] | 0.1 | [0.00,11.67] |
| Percent of patients with respiratory diseases | 0.01 | [0.00,56936.70] | 607.67 | [0.09,4.27e+06] | 0.06 | [0.00,645.24] |
| Percent of patients with HIV | 2.33 | [0.00,4438.35] | 17.97 | [0.08,3863.49] | 0.1 | [0.00,31.08] |
| Percent of prenatal care patients who delivered during the year | 1827.93 | [0.00,2.96e+18] | 1.06E+07 | [0.31,3.59e+14] | 0.00*** | [0.00,0.00] |
| Percent of Medicaid patients | 1.04 | [0.19,5.59] | 0.63 | [0.25,1.58] | 1.21 | [0.47,3.15] |
| ***Primary Care Provider Staffing and Capacity*** |  |  |  |  |  |  |
| PCP Panel Size (Patients Per Provider) | 1 | [1.00,1.00] | 1 | [1.00,1.00] | 1 | [1.00,1.00] |
| *Ratio of nurses to PCP* | 0.89 | [0.57,1.38] | 1.21 | [0.93,1.56] | 0.96 | [0.74,1.25] |
| ***Additional Staffing and Capacity*** |  |  |  |  |  |  |
| Ratio of mental health provider per 5,000 patients | 1.01 | [0.93,1.08] | 1.01 | [0.96,1.06] | 0.98 | [0.93,1.03] |
| *Ratio of dental provider per 2,500 patients* | 1.07 | [0.75,1.53] | 1.1 | [0.90,1.34] | 0.82 | [0.66,1.02] |
| Ratio of enabling service staff per 5,000 patients | 0.99 | [0.95,1.02] | 0.98 | [0.96,1.01] | 1.01 | [0.98,1.03] |
| Average number of services provided in addition to medical care | 0.9 | [0.74,1.09] | 1.05 | [0.96,1.16] | 0.9 | [0.82,1.00] |
| ***Financial Resources*** |  |  |  |  |  |  |
| Per capita total revenues | 1.02 | [0.72,1.43] | 0.78 | [0.60,1.01] | 1.45** | [1.11,1.89] |
| Proportion of total revenues that are from 330 grants | 0.92 | [0.16,5.27] | 0.45 | [0.17,1.22] | 0.58 | [0.20,1.73] |
| ***Contextual characteristics*** |  |  |  |  |  |  |
| Ratio of PCP per 5,000 population in county | 1.24* | [1.04,1.49] | 1.10* | [1.01,1.20] | 1.05 | [0.96,1.15] |
| Proportion below federal poverty guideline in county | 0.96 | [0.92,1.01] | 1 | [0.97,1.03] | 1.03* | [1.00,1.06] |
| Proportion of minority in county | 2.11 | [0.31,14.39] | 0.75 | [0.27,2.05] | 0.41 | [0.14,1.17] |
| Analyses were conducted using logistic regression models. | | | | | | |
| Statistically significant at *p<0.05; **p<0.01; ***p<0.001. | | | | | | |
| BMI, body mass index; CAD, coronary artery disease; IVD, ischemic vascular disease; HIV, human immunodeficiency virus; HbA1c, Hemoglobin A1c; Coef., beta coefficient; CI, confidence interval; OR, odds ratio. | | | | | | |
