# Supplementary material for: Assessing clinical quality performance and staffing capacity differences between urban and rural Health Resources and Services Administration-funded health centers in the United States: A cross sectional study
Source: PLoS One. 2020 Dec 8;15(12):e0242844. doi: 10.1371/journal.pone.0242844 (PMC7723285; doi:10.1371/journal.pone.0242844)
Supplement: S8 Table — (DOCX) [file pone.0242844.s010.docx]

| **S8 Table. Logistic Regression Models of Health Centers that Met Outcome Quality Performance Indicator Benchmarks** | | | | |
| --- | --- | --- | --- | --- |
|  | Met Patients with Diabetes with Hemoglobin A1c Greater Than 9% Performance Measure | | Met Patients with Hypertension with Blood Pressure below 140/90 Performance Measure | |
| **Sample Size** | 1,233 | | 1,233 | |
|  | OR | 95% CI | OR | 95% CI |
| ***Urban*** | 1.73 | [0.52,5.78] | 0.87 | [0.59,1.30] |
| ***Organization Size*** |  |  |  |  |
| Average number of sites | 0.96 | [0.87,1.07] | 1 | [0.98,1.03] |
| Average number of patients seen during the year | 0.82 | [0.48,1.38] | 1.18* | [1.04,1.33] |
| ***Patient Characteristics*** |  |  |  |  |
| Percent of patients that were racial/ethnic minorities | 1.93 | [0.12,30.12] | 0.18** | [0.07,0.51] |
| Percent of patients that spoke with primary care provider (PCP) in a language other than English | 2.16 | [0.20,23.01] | 19.22*** | [7.25,51.00] |
| Percent of patients of patients 65 years and older | 0 | [0.00,35.25] | 135.19* | [2.57,7102.15] |
| Percent of patients between 0--17 years | 0.3 | [0.01,6.75] | 2.58 | [0.60,11.00] |
| Percent of patients with heart related disease | 1.47 | [0.00,3.00e+16] | 76.95 | [0.00,1.42e+07] |
| Percent of patients with diabetes or endocrine diseases | 0 | [0.00,6.32] | 69.4 | [0.49,9829.82] |
| Percent of patients with respiratory diseases | 5.91E+08 | [0.01,3.58e+19] | 99.25 | [0.01,1.78e+06] |
| Percent of patients with HIV | 0 | [0.00,2.10e+14] | 0.61 | [0.00,125.01] |
| Percent of prenatal care patients who delivered during the year | 0 | [0.00,1.12e+06] | 0.05 | [0.00,738517.03] |
| Percent of Medicaid Patients | 4.86 | [0.39,59.86] | 4.20** | [1.60,11.04] |
| ***PCP Staffing and Capacity*** |  |  |  |  |
| PCP Panel Size (Patients Per Provider) | 1 | [1.00,1.00] | 1 | [1.00,1.00] |
| Ratio of nurses to PCP | 1.1 | [0.53,2.29] | 1.09 | [0.83,1.43] |
| ***Additional Staffing and Capacity*** |  |  |  |  |
| Ratio of mental health provider per 5,000 patients | 1.01 | [0.92,1.12] | 0.99 | [0.94,1.05] |
| Ratio of dental provider per 2,500 patients | 0.68 | [0.35,1.30] | 1.2 | [0.97,1.50] |
| Ratio of enabling service staff per 5,000 patients | 0.93 | [0.84,1.04] | 1 | [0.98,1.03] |
| Average number of services provided in addition to medical care | 0.89 | [0.64,1.24] | 0.94 | [0.84,1.04] |
| ***Financial Resources*** |  |  |  |  |
| Per capita total revenues | 1.35 | [0.78,2.32] | 1.17 | [0.93,1.47] |
| Proportion of total revenues that are from 330 grants | 3.81 | [0.31,47.11] | 0.15*** | [0.05,0.42] |
| ***Contextual Characteristics*** |  |  |  |  |
| Ratio of PCP per 5,000 population in county | 0.87 | [0.63,1.18] | 0.99 | [0.90,1.09] |
| Proportion below federal poverty guideline in county | 0.98 | [0.90,1.07] | 0.98 | [0.95,1.01] |
| Proportion of minority in county | 6.07 | [0.35,104.80] | 1.01 | [0.35,2.94] |
| Analyses were conducted using logistic regression models. | | | | |
| Statistically significant at *p<0.05; **p<0.01; ***p<0.001. | | | | |
| BMI, body mass index; CAD, coronary artery disease; IVD, ischemic vascular disease; HIV, human immunodeficiency virus; HbA1c, Hemoglobin A1c ; Coef., beta coefficient; CI, confidence interval; OR, odds ratio | | | | |
