# Supplementary material for: Assessing clinical quality performance and staffing capacity differences between urban and rural Health Resources and Services Administration-funded health centers in the United States: A cross sectional study
Source: PLoS One. 2020 Dec 8;15(12):e0242844. doi: 10.1371/journal.pone.0242844 (PMC7723285; doi:10.1371/journal.pone.0242844)
Supplement: Attachment — Submitted filename: Response to Reviewers-final.docx [file pone.0242844.s011.docx]

| **Reviewer Comment** | **Response to Reviewers** | **Location in Manuscript** |
| --- | --- | --- |
| **Reviewer #1** |  |  |
| Summary: The authors first merged the data collected from 1,373 HRSA-funded Health Centers (HC) and county level data of the 2016 AHRF, and then proceeded to tabulate the various health quality indicators broken down by HC-reported urban/rural status. To discern the adjusted differences, outcome-specific regression models were built (adjusted for patient-level, organizational level, and county-level variables.) The authors found that among adjusted model, HIV linkage persisted to be lower in the rural HCs, while other urban/rural differences were explained away by the various independent variables, with proportions of non-English speakers, people living with diabetes, and people aged 17 or below, as well as PCP/population being the frequent candidates. The authors provided policy-level suggestions on such findings.  Overall, I really enjoyed reading the paper. The data choice was proper and the idea that urban/rural engulfs many different factors was well displayed. The policy-level recommendations were also moderately realistic that they could be referenced by interested policy stakeholders. However, I have some major concerns on result presentation and statistical analyses. I am listing them here for the authors’ reference and I hope addressing them may further strengthen their work. |  |  |
| The results in the supplementary tables should be in the text: First, nearly every statistically significant difference in Table 1 was verbatim mentioned in text, rendering Table 1 unnecessary; Second, Tables 2 and Table 3 are better combined so that the unadjusted and adjusted differences can be more readily compared and contrasted; Third, urban/rural differences in healthcare quality is well-reported but the factors contributing to that in a systematic analysis are not as well-known. If the authors can creatively visualize the significance levels and directions of the regression coefficients without resorting to showing large amount of numbers, then readers will be able to appreciate important interpretations like the one spanning from line 236 through 241. | We have edited description of Table 1 in the Results to reduce length and only emphasize important findings.  We have combined Tables 2 and 3 and reformatted to visualize the significant findings as recommended.  We did not include the coefficients from the supplemental tables in the text. Instead we reported the overall predicted probabilities that show the degree to which a given outcome differs between urban and rural HCs. We then supplemented that discussion with what indicators in models contributed to the outcomes. We believe that it was a more efficient way of describing the results of the extensive models and less confusing to the readers. We added a table that highlighted the significant coefficients for each quality indicator and whether they were positive or negative in response to this comment. | Results, Page 12-13, line 254-263  Revised Table 2 (previously Tables 2 and 3)  Table 3 (new) |
| The use of GLM deserves some clarification: “Generalized Linear Model” includes a lot of different statistical models, so please specify that in the Abstract. From the Methods (Line 189 through 196) it seems the authors set it as a logistic regression, if so, please just state that. Assuming the analysis was indeed logistics regression, why is it the best choice given the dependent variables were continuous percentages? Since there are also beta regression and probit regression, etc. which could be more suitable for these kinds of outcomes, it would be helpful if the authors could provide a method-related paper to justify the use of logistic regression in this fashion. Along the same line, if dichotomization was performed, then please provide the scheme. | The underlying data of the dependent variables are presented as a proportion. For ease of interpretation, the dependent variables are transformed to proportions ranging between zero and one. We used a generalized linear model, not a logistic regression, and have made edits to the Methods to make this clear. We have revised the Methods to make clear the dependent variables are proportions and added detail to the Statistical Analysis section on how we performed this transformation. | Abstract, Page 4, line 69  Methods, Dependent variables, page 9, line 167  Methods, Statistical analysis, Page 11, line 231 |
| How did the model address the difference sizes of HC? The overall analysis seems to assume equal weight for every HC, are they comparable in size (e.g. in terms of patients served)? If not, should the summary statistics and regression models be weighted? | Health centers are not comparable in size and as a result, we have controlled for the average number of sites and patients seen during the year in each model. We have also controlled for a number of patient characteristics. The full regression models with control variables are presented in the Supplemental Appendices. We are using the entire population of health centers in 2017 (N=1,373), not a sample. | S2-S4 Tables |
| Concerns on data management: The attached Excel data set shows that HIV linkage was missing in almost half of the HCs. Given HIV linkage was the only characteristic still found to be different in urban/rural settings, the prevalence of missing outcomes as well as some speculation are merited. In addition, some of the percentage data were shown in percentage, and yet some were shown in fraction (e.g. percent of patient with HIV.) This may explain why the regression coefficients for those covariates in fraction were much larger. I would suggest a round of audit to verify the data, software syntax, and output results. | All clinical quality measure data are displayed as proportions, including HIV linkage to care. We have assessed the missing HIV linkage to care outcomes and found there was a relationship with urban/rural settings. We have added a sentence in the Limitations to address this. We further explain that missingness among rural health centers may be due to low prevalence of HIV and could be too sensitive to report and suggest a conservative interpretation.  We have also confirmed in the software syntax the percentage data displayed in the outcome variables were rescaled as a fraction. | Discussion, page 31, lines 429-435  Methods, Page 9-10, lines 166-167 |
| [Abstract] The starting sentence created a false impression that HCs are exclusive to rural area, creating some confusion later. Please revise. | We have revised this sentence to indicate that health centers are located nationally. | Abstract, Page 4, line 59-62 |
| [Line 115 through 117] The study design, data, and analysis do not support this objective. First, it is cross-sectional so “contribute in reducing disparities” could be over-reaching; second, the analysis adjusted for many causal downstream variables of urban/rural, while I would agree that the work unpacked what urban/rural entails, the fact that the urban/rural became largely statistically non-significant is not indicative of reducing disparity, but perhaps mediation adjustment. | We have revised this sentence to be more conservative and consistent with our study design and analysis. | Introduction, Page 6, line 95-100 |
| [Line 137 through 139] Are there only 15 in HEDIS? If there were more than 15, how did the authors decide on the final list? | There are a total of 15 clinical quality measures that health centers are required to report and we decided to use all 15 measures. These 15 measures have benchmarks in HEDIS. We have revised for clarity. | Methods, Page 8, line 158-163 |
| [Line 194] The brand name should be written as Stata. | Revised as suggested. | Methods, Page 12, line 240 |
| [Line 202] Add SD to the acronym list. | We have added to the acronym list as suggested. | Title Page, Page 3, line 57 |
| [Table 1 and others, including Excel file] Please check the label “Percent of patients of patients 65 years and older.” | Revised for correctness. | Table 1 and S2 Table |
| [Table 3] HEDIS benchmarks should be accompanied by “percent of HCs that exceed the benchmark” rather than using only the sample mean to determine adherence. | We have revised Table 2 to include the proportion of health centers that achieved HEDIS benchmarks. | Revised Table 2 (previously Table 2 and 3)  Methods, Statistical Analysis |
| **Reviewer #2** |  |  |
| This article uses the Uniform Data Set to compare HRSA funded clinics operating in urban and rural areas using a number of standard quality metrics. The authors find that after controlling for confounders, there are no statistically significant differences between urban and rural clinics for most outcome measures. The one exception is that Rural clinics "had lower rates of linking patients newly diagnosed with HIV to care." | No response is required. |  |
| Overall, this is a nice article that makes a useful contribution to the literature. As the authors point out, there are relatively few comparisons of this sort, which is surprising given the well know differences in urban-rural health outcomes. Despite this, I think the paper could benefit from a bit more reflection on both the premise of the study and the implications of these findings. Most studies documenting urban-rural differences in health status point to social determinants as an explanation. To the degree that there is a focus on health care, the usual emphasis is on the availability of care, not quality. Did the authors have reason to believe there might be differences in the quality (as opposed to the quantity) of care available to people living in urban and rural areas? This is an underlying assumption of the analysis that the authors do not set up well. | There is literature that shows differences in quality of care outcomes in the general urban/rural population, as well as other providers. Due to this evidence, we believe in examining urban/rural differences in health center settings in particular. We have added additional sentences in the Introduction in order to address differences in quality. | Introduction, Page 6, line 106-108 |
| Second, I think the quality of care available in HRSA funded clinics is important, but given the relatively small role that such clinics play in the overall health system, would it be reasonable to suggest that differences in quality would be sufficient to explain urban-rural differences in the first place? Or are the authors focused more narrowly on urban-rural differences in health care for populations who are likely to seek care in HRSA clinics and other safety-net organizations? | HCs are the cornerstone of the safety net system and the only providers that provide care to low-income and uninsured patients regardless of income or any other factor. Other providers including public systems organized by counties have various restrictions. In addition, HCs are often the only comprehensive provider of care in rural areas. Therefore, the role of HCs in providing access to high quality care in general and in rural areas in particular is very important.  We are focused on urban/rural differences among populations that are served by HRSA-funded health centers, but our results have implications for quality of care in urban and rural areas more broadly, particularly when HCs are the primary or only providers of care. | Introduction, Page 7, line 123-125 |
| Third, what should policy makers do with this information? If there is a concern about quality differences among HRSA clinics in urban and rural areas, the authors have offered comforting evidence -- but the urban rural health differences remain. So if it is not a quality difference, what's driving the problem? | We cannot explain what explain urban/rural differences in quality from our data. However, we have shown that a subset of rural populations have quality of care on par with urban areas. Therefore, policy efforts should be focused on other providers who are likely to be the source of urban/rural disparities. We have revised the implications to address this point. | Discussion, Page 34, line 479-481 |
| Finally, I think the authors should say a bit more about their HIV finding. There was an article published in the NYT recently arguing that HIV is increasing in rural areas, but that these parts of the country are not ready for it. The findings in this paper are one small part of that, but the findings are certainly consistent with the concerns expressed in the article. I think the authors should put the HIV finding into the larger context of rural public health and health care capacity to address a growing HIV problem in these communities. | We have added additional information to support how health centers have provided additional funding support to address HIV. | Discussion, Page 33, line 458-460 |
