# Supplementary material for: Assessing clinical quality performance and staffing capacity differences between urban and rural Health Resources and Services Administration-funded health centers in the United States: A cross sectional study
Source: PLoS One. 2020 Dec 8;15(12):e0242844. doi: 10.1371/journal.pone.0242844 (PMC7723285; doi:10.1371/journal.pone.0242844)
Supplement: Attachment — Submitted filename: Urban Rural CQM Response to Reviewers RR2-final.docx [file pone.0242844.s012.docx]

| **Reviewer Comment** | **Response to Reviewers** | **Location in Manuscript** |
| --- | --- | --- |
| **Reviewer #1** |  |  |
| Major comments: Thank you for addressing most of my comments concerning the presentation of the results. Tables 2 & 3 appear informative and I hope readers will find the results more readily digestible.  There are a few remaining issues that the authors did briefly responded to and I would like to raise them again:  It appears that the GLM analysis was based on binomial family and logit link, which makes this a logistic regression. After reading the authors’ response, my curiosity remains unsated and I wish the authors will address this explicitly: why was logistic regression used on a continuous variable that is bound between 0 and 1? It would be great if the author can cite a technical document that this is indeed a correct approach. The fractional dependent variables (like pct_diabete) look very much like a bell curve and I couldn’t wrap my head around using binomial. If we tried to use the Stata command “logit” or “logistic” to repeat this analysis we may also find that Stata would in fact display a warning and refuse to proceed. Please also consider if fracreg (Fractional response regression) function in Stata would be a more appropriate candidate. | We used GLM regressions for the analyses because the dependent variables are a proportion bound between 0 and 1 and therefore had both a lower and upper bound ceiling. The following citation supports our rationale for using the GLM analysis (Baum CF. Stata Tip 63: Modeling Proportions. The Stata Journal. 2008;8(2):299-303. doi:10.1177/1536867X0800800212). In response to the reviewer’s comment we changed the regressions to fracreg even though our tests indicated the two models yielded the exact same results. We included a technical citation to support use of fracreg in the manuscript. | Methods, Page 11, line 209-210 |
| With the Excel data, I was able to check some statistics in Table 1. It seems the data remained unweighted. Given the mean (SD) patient of 19,792 (23,663), there is a huge variability among the sizes. If a HC serving 1,000 patients has and indicator at 0%, and a HC serving 20,000 patients has it at 50%, would the mean be closer to 25.0% or 47.6%? It’d be great if the authors can justify why they favored the unweighted approach. | We did not weight the data because the goal of this manuscript was to compare the performance of HC in urban and rural locations. For this goal, every HC would have to exert the same amount of influence on the results. As the reviewer points out, the weighted data would allow larger HCs to exert more of an influence on the results. That analyses would be more appropriate if our aim was to compare the population-level impact.  To illustrate the difference between weighted and unweighted data, we conducted weighted regressions to assess how the results change. We found the majority of coefficients had similar effects in both types of analyses, with the exception of HIV linkage to care and low birthweight. In weighted analyses, HIV linkage to care was no longer significant, but the direction of association did not change. In the weighted model, low birthweight became significant but the direction of the associations did not change. | No change. |
| **Reviewer #3** |  |  |
| The analyses in this manuscript appear to be done well. I had some suggestions below to strengthen the descriptions and, potentially, improve the results. I think the biggest problem is the manuscript is so dense. Table 2 and the results section have quite a bit of information. I have a couple suggestions for this in my comments which may or may not help. | We thank the reviewer for their comments. We have added Figure 1 to visually display the significant differences in performance measures between urban and rural HCs. We did not include results that were not significant to reduce the size of this figure. We moved the original Table 2 into the appendix to all performance measures . | Figure 1 (new)  Table 2 |
| 1. (line 172) This statement is a little confusing since an indicator variable is 0 or 1 and not a proportion. I'm guessing created indicator variables for each of the 15 DVs and then created a proportion from those? That would fit with your methods. | We have removed this sentence to reduce confusion. The dependent variables are indeed proportions bound between 0 and 1 and therefore had both a lower and upper bound ceiling. They indicate the proportion of patients that had received specific services or had a specific outcome. The methods sections are revised in response to both reviewers’ comments. | Methods, Page 8, line 158 |
| 2. (lines 205-206) I think this choice of method is good. I suggest including a methodological citation for the method, probably McCullagh and Nelder's book will be fine. I think the main question is what your residuals look like and whether there is potential for systematic over- or under-fitting. My guess is that your covariates have eliminated this, but it would be good for you to check this. It may be that adding random effects by state to account for any state-to-state differences could be helpful. Another thought were spatial random effects, but my guess is that the urban/rural variable will be good enough for that. | As indicated in the previous responses to similar comments above, we are now using fracreg or fractional outcome regression, which produced the same results as GLM and have added a citation to support the use of this regression model.  We have also checked the residual plots for all our models and found that there was no evidence of model under- or overfitting.  We have controlled for county-level variations using county-level characteristics, as well as the urban/rural variable as the reviewer mentioned. We have not included random effects by state to avoid any potential overfitting/overcontrolling. | Methods, Page 11, lines 209-210 |
| 3. (line 210) How many or what percentage of HCs were dropped due to missing data? Rules of thumb vary on when complete case analyses are still valid. My suggestion is if > 5% are dropped, then start exploring the missing data. If > 15% are missing, then you'll probably need to do something about it, e.g., multiple imputation. | We examined the proportion of missing for each dependent variable and found that no variables were missing by greater than 5%. We did not impute the dependent variables with missing values following recommendation from Hippel (Von Hippel, P.T. (2007), REGRESSION WITH MISSING YS: AN IMPROVED STRATEGY FOR ANALYZING MULTIPLY IMPUTED DATA. Sociological Methodology, 37: 83-117. doi:[10.1111/j.1467-9531.2007.00180.x](https://doi.org/10.1111/j.1467-9531.2007.00180.x)  ). | No change |
| 4. (line 211) Sorry, I'm not a Stata user. Does the margins command produce confidence intervals or prediction intervals? | The margins command produces predicted probabilities with confidence intervals. | S2 Table |
| 5. Did you perform any variable selection or were all the IVs included in all analyses? | We had selected independent variables that could influence HC performance conceptually and included all of them in the models to avoid omitted variable bias. | No change |
| 6. (Table 2) This table is quite dense and I wonder if you thought about moving some of these results to a figure, especially the predicted probabilities. It might make for a nice visual and could make table 2 more readable. I also wondered if there was any way to abbreviate the measures in the first column. | We agree that the table was dense. This was the result of requests from previous reviewers to present the data in that way. We have now presented some of the data in the original Table 2 into Figure 1 as suggested and abbreviated the name of the measures for better readability. We then adjusted the other tables including S2 Table and Table 2 still reports probabilities of met or exceeding benchmarks. | Figure 1 (new)  S2 Table  Table 2 |
| 7. (Table S2) I am not sure I understand the coefficients in this table. Are they the raw coefficient values from the binomial models? If so, they should be exponentiated to become odds ratios and reported as such. | Yes, the supplemental data displayed beta coefficients. We have now revised them to report odds ratios as suggested. | S3 Table- S5 Table |
